# Ibuprofen Treatment Reduces the Neuroinflammatory Response and Associated Neuronal and White Matter Impairment in the Growth Restricted Newborn

**DOI:** 10.3389/fphys.2019.00541

**Published:** 2019-05-10

**Authors:** Julie A. Wixey, Kishen R. Sukumar, Rinaldi Pretorius, Kah Meng Lee, Paul B. Colditz, S. Tracey Bjorkman, Kirat K. Chand

**Affiliations:** ^1^UQ Centre for Clinical Research, Faculty of Medicine, The University of Queensland, Brisbane, QLD, Australia; ^2^Institute of Health Biomedical Innovation, Queensland University of Technology, Brisbane, QLD, Australia; ^3^Perinatal Research Centre, Royal Brisbane and Women’s Hospital, Brisbane, QLD, Australia

**Keywords:** placental insufficiency, growth retardation, fetal growth restriction, astrocytes, microglia, ibuprofen, newborn brain injury

## Abstract

Intrauterine growth restriction (IUGR) is a condition where the fetus does not achieve optimal growth, commonly caused by placental insufficiency. The chronic decrease in blood flow restricts oxygen and nutrient supply to the fetus, which can damage numerous organ systems, with the fetal brain being particularly vulnerable. Although white matter and neuronal injury are evident in IUGR infants, the specific mechanisms underlying these changes are poorly understood. Inflammation is considered to be a main driver in exacerbating brain injury. Using a spontaneous piglet model of IUGR, we aim to determine whether administration of the anti-inflammatory drug ibuprofen will decrease inflammation at postnatal day 4 (P4). The treatment group received ibuprofen (20 mg/kg/day on day 1 and 10 mg/kg/day on days 2 and 3) in piglet formula during the morning feed each day and brains examined on P4. Markers of inflammation, apoptosis, cell proliferation, neuronal injury, and white matter injury were examined. Ibuprofen treatment ameliorated the increase in numbers of microglia and astrocytes in the parietal cortex and white matter tracts of the IUGR piglet brain on P4 as well as decreasing proinflammatory cytokines. Ibuprofen treatment prevented the reduction in apoptosis, neuronal cell counts, and myelin index in the IUGR piglets. Our findings demonstrate ibuprofen reduces the inflammatory response in the IUGR neonatal brain and concurrently reduces neuronal and white matter impairment.

## Introduction

Intrauterine growth restriction (IUGR) is a major pediatric concern associated with increased perinatal mortality and long-term morbidity. The fetal brain is particularly vulnerable to prolonged IUGR conditions with neuronal and white matter disturbances observed in clinical imaging studies (Tolsa et al., 2004; Esteban et al., 2010; Padilla et al., 2015). Large follow up studies on IUGR infants have shown IUGR is associated with neurodevelopmental disabilities including lower academic performance, short-term memory deficits, and attention deficit disorders (Geva et al., 2006; Heinonen et al., 2010). With term born IUGR newborns having a 4- to 7-fold increase of developing cerebral palsy (Jarvis et al., 2003; Jacobsson et al., 2008). Currently there is no treatment available to minimize long-term adverse neurological outcomes in IUGR newborns. It is important to target key mechanisms of brain injury in the IUGR newborn to reduce or prevent long-term neurological dysfunction in these infants.

There is growing evidence of the critical role neuroinflammation plays in IUGR brain injury (Wixey et al., 2017, 2018). Recent studies in animal models of IUGR report an inflammatory response in the IUGR brain (Olivier et al., 2005, 2007; Tolcos et al., 2011; Campbell et al., 2012; Black et al., 2015; Castillo-Melendez et al., 2015; Pham et al., 2015; Rideau Batista Novais et al., 2016; Wixey et al., 2019). Neuroinflammation envelops a set of processes which include increased number of activated microglia, astrogliosis, increased production of proinflammatory cytokines such as interleukin-1β (IL-1β) and tumor necrosis factor-α (TNF-α), and decreased production of anti-inflammatory cytokines (Cai et al., 2006; Kremlev et al., 2007; Carty et al., 2008; Leonardo et al., 2008; Huang et al., 2009; Wixey et al., 2009, 2011a). In the newborn IUGR piglet a robust increase in inflammatory mediators such as activated microglia, astrocytes, and proinflammatory cytokines are associated with neuronal and white matter impairment (Wixey et al., 2019). Worsening brain injury is associated with increases in proinflammatory cytokines in a fetal IUGR guinea pig model (Guo et al., 2010). Furthermore, an increase in proinflammatory cytokines have been reported in the blood of IUGR infants (Mcelrath et al., 2013) and systemic inflammation in the small for gestational age infant is associated with adverse neurodevelopment at 2 years (Leviton et al., 2013). The opportunity to target inflammation to potentially reduce this brain impairment is appealing. As IUGR is detected around birth in majority of cases (Sovio et al., 2015), postnatal neuroprotective therapies may be invaluable. With inflammation present in the IUGR neonatal brain, an anti-inflammatory intervention may reduce adverse effects in the IUGR newborn.

Ibuprofen is a non-steroidal anti-inflammatory drug (NSAID) with anti-inflammatory, anti-pyretic and analgesic properties. It is currently safely used in the preterm neonate to treat patent ductus arteriosus. Ibuprofen acts to inhibit cyclooxygenase 1 and 2 (COX-1 and -2) activity, that have various critical functions through production of prostaglandins, in regulating blood flow as well as inflammatory pathways. Ibuprofen has been shown to inhibit neuroinflammation and have neuroprotective effects in neonatal animal models of acute hypoxia-ischemia (HI) (Carty et al., 2011; Wixey et al., 2012). One week treatment of ibuprofen attenuates HI-induced increases COX-2 levels, proinflammatory cytokine levels and microglial activation in the neonatal HI rat brain (Wixey et al., 2012) whilst concurrently protecting the white matter and neurons from injury (Carty et al., 2011; Wixey et al., 2012). However, the potential neuroprotective effects of ibuprofen treatment for the IUGR newborn have not been examined in associated animal models.

In the present study we hypothesized that ibuprofen treatment can reduce inflammation, as well as neuronal and white matter impairment in the IUGR newborn. Using the preclinical piglet model of IUGR we assessed whether 3 days of oral ibuprofen treatment to the newborn IUGR piglet could not only reduce the neuroinflammatory response, but reduce cell death and neuronal and white matter impairment.

## Materials and Methods

Approval for this study was granted by The University of Queensland Animal Ethics Committee (MED/UQCCR/132/16/RBWH) and was carried out with respect to the National Health and Medical Research Council guidelines (Australia) and ARRIVE guidelines.

Newborn large white piglets (*n* = 24; <18 h) were collected from the UQ Gatton Piggery monitored and cared for at the Herston Medical Research Centre (HMRC) until day of euthanasia on postnatal day 4 (P4). Litter matched pairs were obtained from multiple sows (*n* = 10). Piglets were divided into 4 groups: normally grown (NG) (*n* = 6), IUGR (*n* = 6), NG + ibuprofen (*n* = 6) and IUGR + ibuprofen (*n* = 6); with equal males and females in each group. IUGR piglets were defined by birth bodyweight (<10th percentile on the day of birth) and confirmed by brain to liver weight ratio (B:L) ≥ 1 at postmortem (Bauer et al., 1998; Cox and Marton, 2009; Kalanjati et al., 2017). B:L is used to define asymmetric growth restriction in the IUGR newborn. The IUGR piglet mimics many human outcomes associated with IUGR including asymmetrical growth restriction with brain sparing (Bauer et al., 2003). Inadequate fetal growth in pigs is caused by placental insufficiency (Bauer et al., 2003) which is the most common cause of IUGR in the human population. Therefore, data obtained from the piglet model translates well to the human IUGR. Ibuprofen treatment groups received 20 mg/kg/day on day 1 and 10 mg/kg/day on days 2 and 3. This dosage is routinely used in the human preterm newborn to treat patent ductus arteriosis (Ohlsson et al., 2015). Ibuprofen was mixed with pig milk formula and delivered via an oro-gastric tube at 9 am each morning. On P4, piglets were euthanized via an intracardiac injection of sodium phenobarbital (650 mg/kg; Lethabarb, Virbac, Australia). Brain tissue was collected, weighed, hemisected and coronally sliced. The right hemisphere sections were immersion fixed in 4% paraformaldehyde as previously described (Kalanjati et al., 2011). The parietal cortex from the left hemisphere was snap frozen in liquid nitrogen and stored at −80°C for mRNA analysis.

### Quantitative Polymerase Chain Reaction (qPCR)

RNA was isolated and purified using an RNeasy Tissue Mini Kit (Qiagen) from 30 mg parietal cortex. RNA yield and quality was determined using a NanoDrop spectrophotometer (ND-1000 system). A reverse transcription kit (RT^2^ First Strand Kit; Qiagen) was used for cDNA synthesis. Synthesized cDNA was pooled for each group giving equal concentrations from each animal in the pooled sample. The pooled synthesized cDNA was combined with RT^2^ SYBR Green qPCR Mastermix (Qiagen) and loaded into the Pig Inflammatory Cytokines & Receptors RT^2^ Profiler^TM^ PCR Array (Qiagen, Hilden, Germany). The qPCR reactions were performed using a Qiagen Rotor-Gene Q real-time cycler [10 min at 95°C, 40 cycles (15 s at 95°C; 1 min at 60°C)]. The amplified transcripts were quantified with the comparative CT method using actin, gamma 1 (ACTG1) mRNA expression levels for normalization. The same CT threshold value was used across all arrays to allow comparison between runs.

### Immunohistochemistry

Brain sections from the parietal cortex of the right hemisphere (Pig stereotaxic map, A 5.5 mm; Felix et al., 1999) were embedded in paraffin and coronally sectioned 6 μm apart. Sections were affixed to Menzel Superfrost Plus adhesive slides and air-dried overnight at 37°C. All sections were dewaxed and rehydrated using standard protocols followed by heat induced epitope retrieval using 10 mM citrate buffer of pH 6 at 80°C for 10 min before cooling to room temperature (RT). A hydrophobic barrier was drawn around the tissue followed by non-specific blocking with 5% donkey serum in PBS with 0.5% Triton-X 100 for 1 h at RT. Cellular markers examined using immunohistochemistry were astrocytes [glial fibrillary acidic protein (GFAP); 1:1000, Z0334, Dako], microglia (ionized calcium binding adaptor molecule-1; Iba-1; 1:1000, ab5076; Wako Chemicals), neurons (NeuN; 1:1000; ab177487; Abcam Cambridge), proliferating cells (Ki67; 1:200, ab15580; Abcam) and apoptotic cells (cleaved Caspase-3; 1:500, #9661; Cell Signaling). Primary antibodies were incubated at 4°C for 20 h. Slides were washed in Tris-Buffered Saline followed by incubation with species-specific secondary fluorophores at RT for 1 h (Alexafluor 488, Alexafluor 568; 1:1000, Molecular Probes, Invitrogen Australia, Mount Waverley, VIC, Australia). Tissue was then washed, counterstained with 4′,6-diamidino-2-phenylindole (DAPI), and mounted with Prolong Gold antifade (Molecular Probes, Invitrogen Australia, Mount Waverley, VIC, Australia). Negative control sections without primary antibodies were processed in parallel. Staining was conducted in triplicates for all animals.

#### Luxol Fast Blue Staining

General myelination status of IUGR brains was assessed using Luxol Fast Blue (LFB) staining as previously described (Wixey et al., 2019). Tissue sections underwent standard dewaxing and rehydration followed by overnight immersion in LFB solution at 57°C. Sections were immersed in 95% ethanol and differentiated in 0.05% lithium carbonate followed by 70% ethanol until gray and white matter could be distinguished and nuclei decolorized. Tissue was processed and stained simultaneously to minimize variability of LFB staining.

### Image Acquisition and Analysis

Analysis of immunolabeled sections was performed using Olympus BX41 light microscope with a DP70 camera. Pictomicrographs (881.2 μm × 663.5 μm) of gray matter (parietal cortex) and white matter [intragyral white matter (IGWM); subcortical white matter (SCWM); periventricular white matter (PVWM)] were captured for analysis. Four pictomicrographs were captured in each respective area for each animal in triplicate. All tissue was imaged and analyzed under blind conditions by KKC, JAW, KS and RP, and manual counts for NeuN, Caspase-3, Ki67, and Iba-1 were performed.

Microglia were manually counted with respect to morphology in cortical gray matter, IGWM, SCWM, and PVWM of the parietal cortex. Astrocytes in the WM were quantified using densitometry by thresholding the intensity of GFAP labeling using ImageJ (Image Processing and Analysis in Java; National Institutes of Health, Bethesda, MD, United States). Areal density was expressed as percentage of the whole WM for each region covered as previously described (Wixey et al., 2019). For LFB staining, slides were scanned using a Pannoramic SCAN II digital slide scanner (3D HISTECH, Ltd.) with a 20x Plan-apochromat objective and analyzed as previously reported (Wixey et al., 2019). In short, images were converted to high resolution 8-bit grayscale images and thresholded to determine staining intensity from 0 to 127 (0, white; 255, black). This range was divided into quartiles and the percent area stained (% area) for each was calculated. The median gray level of each quartile (14.5, 46.0, 78.5, and 111.0) was then multiplied by % area/100 in each quartile, to give the total myelin index.

### Statistical Analysis

Two-way ANOVA with the *post hoc* Sidak analysis was used to determine differences between NG and IUGR animals under non-treated and ibuprofen-treated conditions (GraphPad Prism 7.0 software, San Diego, CA, United States). Results were expressed as mean ± SEM with statistical significance accepted at *p* < 0.05.

## Results

Mean body weight was significantly lower in both IUGR untreated piglets (*p* < 0.0001) and IUGR ibuprofen treated piglets (*p* < 0.0001) compared to untreated NG piglets (Table 1). There was no significant difference in body weight between the treated and untreated NG piglets (*p* = 0.246). Brain weight was significantly reduced only in the IUGR untreated group compared to the NG group (*p* = 0.018). The mean brain to liver weight ratio was significantly higher in both treated (*p* < 0.0001) and untreated IUGR piglets (*p* < 0.0001) compared to NG piglets indicating asymmetric growth restriction in the IUGR piglets. A small number of the NG ibuprofen treated piglets may have been slightly growth restricted due to the significant difference in liver weight in comparison to NG piglets (*p* = 0.003), however the mean brain to liver weight ratio was <1 for the NG ibuprofen treated group and not significantly different to NG piglets (*p* = 0.269; Table 1).

**Table 1:** Piglet bodyweight, brain weight, and liver weight.

	NG (*n* = 6)	IUGR (*n* = 6)	IUGR+ibuprofen (*n* = 6)	NG+ibuprofen (*n* = 6)
Bodyweight in grams (mean ± SEM)	1973 ± 157.4	^†††^866.7 ± 77.23^∗∗∗∗^	^†††^1000 ± 39.67^∗∗∗∗^	1643 ± 125.6
Brain weight in grams (mean ± SEM)	32.19 ± 1.10	^†^28.66 ± 0.63^∗^	30.18 ± 0.49	32.29 ± 0.59
Liver weight in grams (mean ± SEM)	64.41 ± 4.89	^†††^19.1 ± 1.26^∗∗∗∗^	^††^22.69 ± 2.09^∗∗∗∗^	43.44 ± 4.76^∗∗^
Brain:liver ratio (mean ± SEM)	0.51 ± 0.03	^†††^1.537 ± 0.11^∗∗∗∗^	^††^1.383 ± 0.12^∗∗∗∗^	0.797 ± 0.10

*Mean body weight was significantly lower in both IUGR untreated and ibuprofen treated piglets compared to untreated NG piglets. Brain weight was significantly reduced only in the IUGR untreated group compared to the NG group. The mean brain to liver weight ratio was significantly higher in both treated and untreated IUGR piglets compared to NG piglets indicating asymmetric growth restriction in the IUGR piglets. Values are the mean ± SEM. ^∗^p < 0.05; ^∗∗^p < 0.01; ^∗∗∗∗^p < 0.0001 versus NG. ^†^p < 0.05; ^††^p < 0.01; ^†††^p < 0.001 versus NG+ibuprofen.*

### Inflammatory Response Following Ibuprofen Treatment in the IUGR Piglet Brain

**FIGURE 1:**
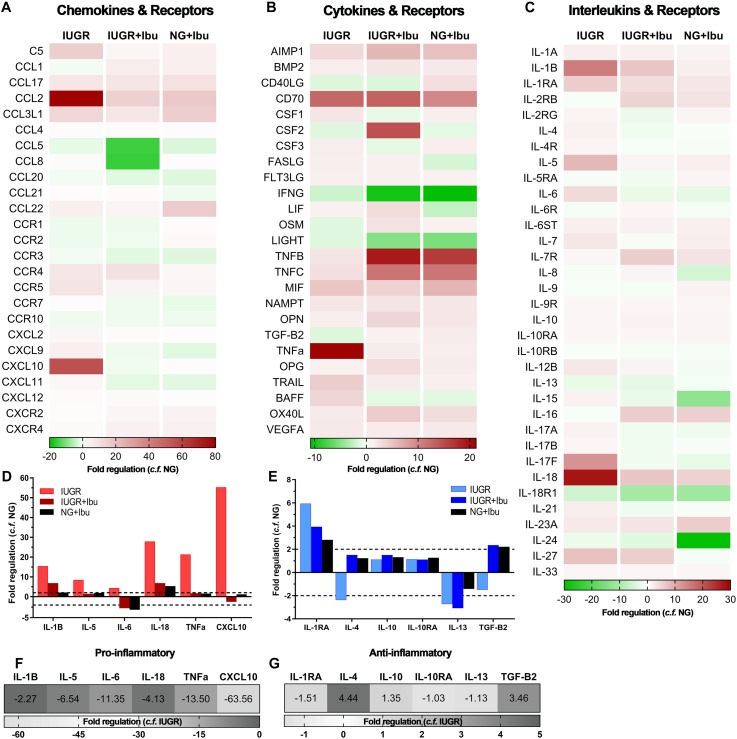
Ibuprofen treatment attenuates expression of inflammatory mediators in IUGR piglet brains. Heat maps of inflammatory profiler arrays separated into chemokines and receptors **(A)**, cytokines and receptors **(B)**, and interleukins and receptors **(C)**. Heat maps demonstrate altered expression of pro- and anti-inflammatory mediators in the cortex of IUGR relative to untreated NG. Treatment with ibuprofen largely down-regulated inflammatory genes relative to untreated NG. Expression of well-characterized pro-inflammatory **(D)** and anti-inflammatory **(E)** genes relative to NG. Ibuprofen treatment in IUGR resulted in down-regulation of pro-inflammatory **(F)** and up-regulation of key anti-inflammatory genes **(G)** when compared with untreated IUGR.

We observed altered expression of both pro- and anti-inflammatory cytokines in the IUGR parietal cortex compared with NG piglets using a PCR array panel of 84 inflammatory genes (Figure 1). Heat maps of fold regulation expression levels of chemokines, cytokines and interleukins compared with NG piglets are represented in Figure 1A–C. The proinflammatory mediators IL-1β, IL-5, IL-6, IL-18, TNFα, and C-X-C motif chemokine (CXCL10) showed high upregulation of expression in the IUGR brain when compared with NG (Figure 1D). Anti-inflammatory markers IL-4 and TGF-β displayed decreased expression in the IUGR brain compared to NG (Figure 1E). Following ibuprofen treatment a reduction in the fold expression of proinflammatory mediators IL-1β (−2.27), IL-5 (−6.54), IL-6 (−11.35), IL-18 (−4.13), TNFα (−13.50), and CXCL10 (−63.56) were evident in IUGR treated piglets compared to IUGR untreated piglets (Figure 1F). Ibuprofen treatment also ameliorated the reductions in the anti-inflammatory cytokines IL-4 (4.44) and TGF-β (3.46) (Figure 1G).

**FIGURE 2:**
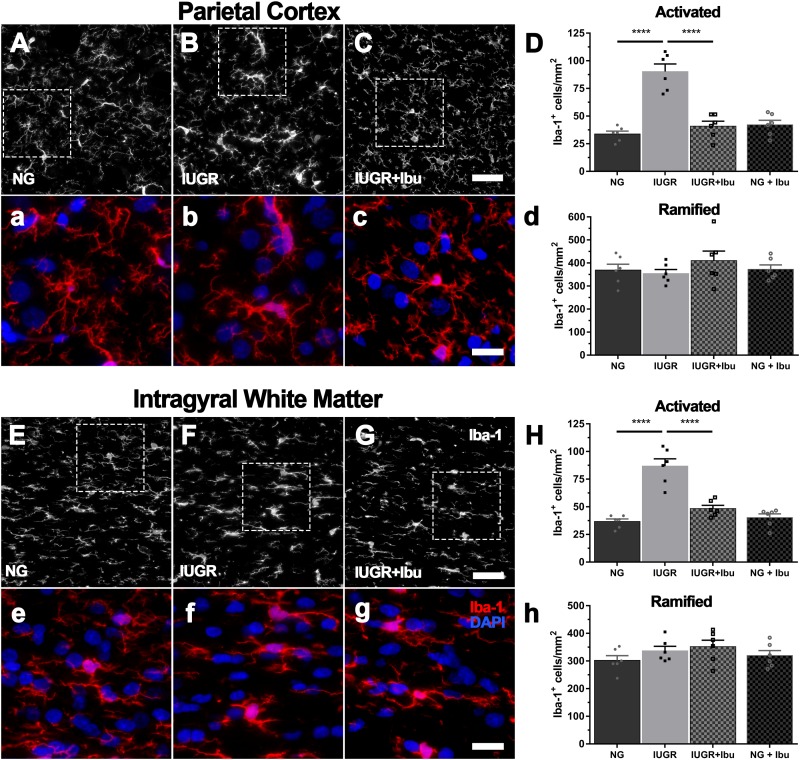
Increased microglial activation in cortex and WM of IUGR. Representative immunofluorescent images of microglial immunolabeling in the PC **(A–C)** and IGWM **(E–G)** of untreated NG, IUGR, and treated IUGR brains. Microglia in untreated IUGR brains displayed dense cell bodies with truncated and thickened processes in both the PC **(B)** and IGWM **(F)**, indicative of microglial activation. NG brains presented microglia with predominantly ramified morphology with long thin extended processes (NG; **A,E**). Quantification based on morphology found significantly higher activated microglia in IUGR compared with NG in both the PC and IGWM (**D,H**; respectively) and no difference in the number of ramified microglia **(d,h)**. Ibuprofen treated IUGR **(C,G)** animals displayed morphology similar to that observed in untreated NG. IUGR animals had significantly lower activated microglial counts when compared with untreated IUGR in both the PC and IGWM **(D,H)**. Scale bars for representative images are 50 μm **(A–C,E–G)** and 20 μm **(a–c,e–g)**. For **(D,H)** *n* = 6 per group. Values are presented as mean ± SEM. Analysis was performed using Two-way ANOVA with Sidak *post hoc* test (^∗∗∗∗^*p* < 0.0001).

Iba-1-positive microglia in NG brains displayed light cell bodies with fine extended processes indicative of a resting state (Figure 2a). In comparison, many of the Iba-1-positive microglia in IUGR brains resembled the morphology of activated microglia with darker cell bodies and thickened retracted processes as previously described (Wixey et al., 2019). When the Iba-1-positive cells were counted based on morphology, a significant increase in activated microglia was evident in the parietal cortex (62.6%; *p* < 0.0001), IGWM (57.7%; *p* < 0.0001), SCWM (40.0%; NG: 44.85 ± 4.64; IUGR: 75.14 ± 2.96; *p* = 0.0006) and PVWM (49.7%; NG: 48.88 ± 4.23; IUGR: 97.27 ± 2.46; *p* < 0.0001) of IUGR piglets compared to NGs (Figure 2). No significant difference in numbers of Iba-1-positive ramified (resting) microglia were observed in any of the regions examined in IUGR piglets compared to NGs (Figure 2). Figure 2 graphs demonstrating parietal cortex and IGWM only as SCWM and PVWM results are similar. Data reported above.

Ibuprofen treatment significantly alleviated the increase in Iba-1-positive activated microglia in the parietal cortex (54.7%; *p* < 0.0001), IGWM (44.2%; *p* < 0.0001), SCWM (39.5%; IUGR 75.14 ± 2.96; IUGR+Ibu: 45.68 ± 4.87; *p* = 0.0008), and PVWM (52.4%; IUGR: 97.27 ± 2.46; IUGR+Ibu: 46.26 ± 4.02; *p* < 0.0001) in treated IUGR piglets compared to untreated IUGR piglets (Figure 2). Furthermore, the Iba-1-positive microglial morphology in the ibuprofen treated IUGR piglets resembled that of healthy microglia in NG piglets with the processes appearing finer and less dense (Figure 2h). Ibuprofen treatment did not affect the numbers of Iba-1-positive ramified microglia through all regions examined.

**FIGURE 3:**
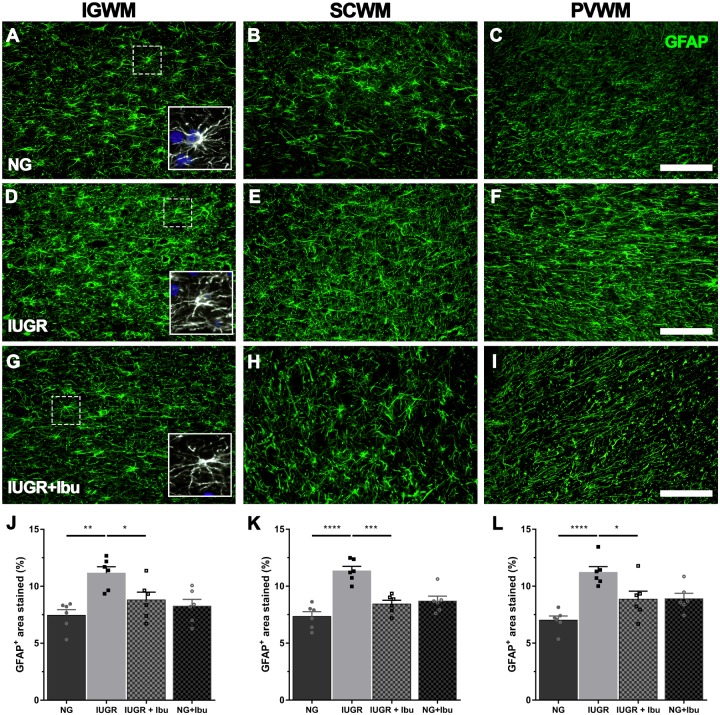
Ibuprofen treatment prevents astrogliosis in white matter regions of IUGR brain. **(A)** Representative images of astrocytes labeled with GFAP in the intragyral (IGWM; first column), subcortical (SCWM; second column) and periventricular (PVWM; third column) white matter regions in P4 NG **(A–C)**, IUGR **(D–F)**, and Ibuprofen treated IUGR (IUGR+Ibu; **G–I**) brains. Astrocytes in NG displayed long extended processes and small cell bodies, typical of ramified astrocytes (see inserts; **A–C**). GFAP staining in IUGR white matter more abundant and showed morphology with enlarged cell bodies and thickened processes (see inserts **D–F**). Ibuprofen treated IUGR displayed ramified morphology (see inserts; **G–I**). Scale bar is 100 μm. Quantification of GFAP expression using densitometry showed an increase in GFAP expression in all white matter regions investigated IUGR when compared with NG brains (**J**, IGWM; **K**, SCWM; **L**, PVWM). Ibuprofen treated IUGR showed significantly lower GFAP density in comparison to untreated IUGR in all regions. No significant alterations in GFAP expression were observed in NG treated animals. For **(J–L)** *n* = 6 for all groups. Values are presented as mean ± SEM. Two-way ANOVA with Sidak *post hoc* test (^∗^*p* < 0.05; ^∗∗^*p* < 0.01; ^∗∗∗^*p* < 0.001; ^∗∗∗∗^*p* < 0.0001).

Glial fibrillary acidic protein-positive astrocytes were observed through the WM tracts of the parietal cortex. In the NG piglet brain GFAP-positive cells demonstrated multiple long branching processes from the cell body typical of normal astrocyte morphology. In the IUGR piglet brains many of the GFAP-positive astrocytes displayed morphology of a more reactive state with retracted processes and large cell bodies (Figure 3). GFAP-positive astrocyte density was significantly increased in the IUGR IGWM (33.2%; *p* = 0.0013), SCWM (35.3%; *p* < 0.0001) and PVWM (37.5%; *p* < 0.0001) compared to NGs (Figure 3J–L). Ibuprofen treatment alleviated the GFAP-positive astrocyte increase in the IGWM (21.2%; *p* = 0.0460), SCWM (25.7%; *p* = 0.0002), and PVWM (21.0%; *p* = 0.0229) in IUGR treated piglets compared to untreated IUGR piglets (Figure 3). There was no significant difference between treated IUGR and NG piglets in any of the regions examined.

### Myelination Status in White Matter in IUGR Piglet Brain Following Ibuprofen Treatment

**FIGURE 4:**
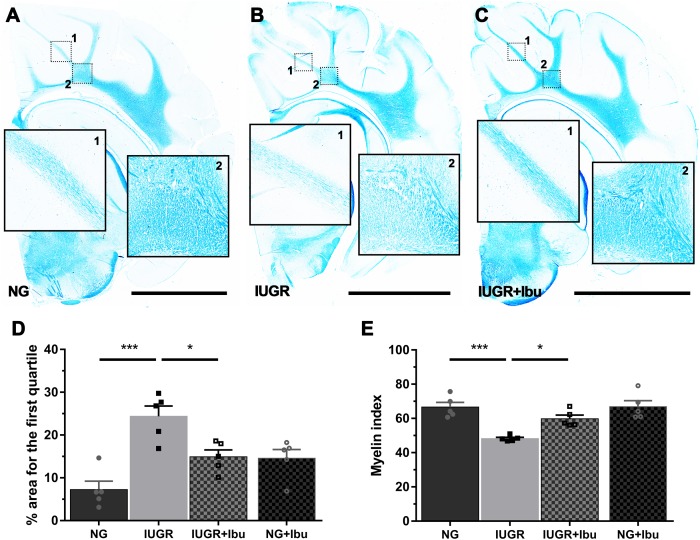
Ibuprofen treatment improves myelination status in white matter of IUGR piglets. Representative images demonstrating high expression of myelin stained with Luxol fast blue (LFB) in untreated NG **(A)** compared with IUGR **(B)** brains (scale bar = 10 mm). The degree of LFB staining was consistent across all white matter (WM) regions in NG brains, while IUGR displayed overall lower levels with sparse staining evident across WM regions (compare B1&2 with A1&2). **(C)** Ibuprofen treated IUGR showed staining consistent with that observed in NG. **(D)** IUGR brains presented greater areas with significantly lower LFB staining, as indicated by percentage area for the first quartile. This was attenuated in IUGR treated with Ibuprofen. **(E)** IUGR displayed a decreased myelin status as shown by the lower myelin index in IUGR compared with NG. Ibuprofen treated animals maintained myelin status consistent with untreated NG. For **(D,E)** *n* = 5 for each group. Values are presented as mean ± SEM. Two-way ANOVA with Sidak *post hoc* test (^∗^*p* < 0.05; ^∗∗∗^*p* < 0.001).

Using Luxol Fast Blue (LFB) to observe myelination we observed organized white matter fiber tracts through the IGWM, SCWM, and PVWM in the NG piglet brains. In the IUGR piglet brain these tracts show lower staining intensity and regions with sparse staining (Figure 4). IUGR brains demonstrate significantly higher areas of WM in the first quartile (low intensity LFB staining), as indicated by the % area for the first quartile, when compared with NG (Figure 4D; *p* = 0.001). Furthermore, there was a significant reduction in myelin index in the IUGR piglet brain compared to NG (Figure 4E; *p* = 0.0005). Ibuprofen treatment ameliorated all of these disruptions in the IUGR piglet brain (Figure 4D,E), with white matter tracts displaying dense LFB staining similar to the myelin tracts in the NG piglet brain.

### Neuronal Integrity in the IUGR Piglet Brain Following Ibuprofen Treatment

**FIGURE 5:**
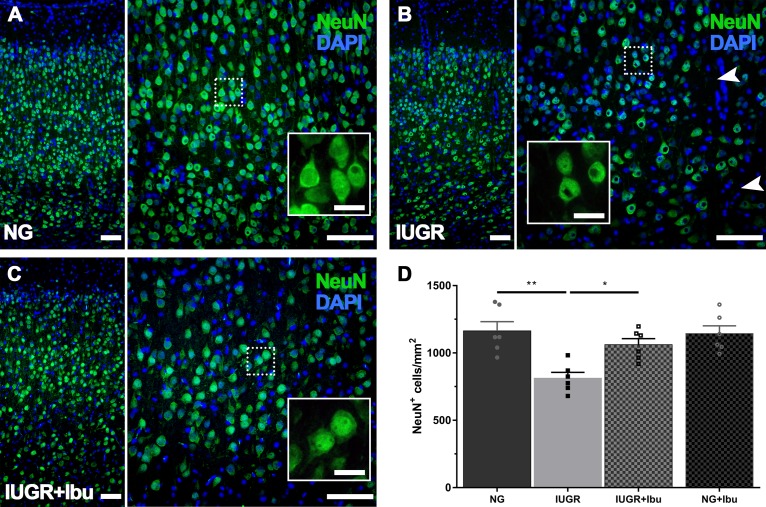
Recovered expression of mature neurons following Ibuprofen treatment in IUGR brains. **(A)** Representative immunofluorescent staining of mature neurons using the neuron-specific nuclear marker NeuN (green) in the parietal cortex. NG brains displayed robust NeuN expression throughout the parietal cortex with large plump neurons (see insert). In comparison IUGR brains **(B)** showed regions void of neurons (see arrowheads), and altered NeuN immunolabeling (insert). Ibuprofen treated IUGR show similar staining patterns to untreated NG **(C)**. Scale bars = 100 μm for lower magnification images and 50 μm for high magnification inserts. **(D)** Quantification of cell counts found a decreased number of NeuN-positive labeled cells in IUGR compared with NG brains. IUGR+Ibu animals had higher NeuN-positive cells compared with untreated IUGR. For **(D)** *n* = 6 for each group. Values are presented as mean ± SEM. Two-way ANOVA with Sidak *post hoc* test (^∗^*p* < 0.05; ^∗∗^*p* < 0.005).

Using immunohistochemistry, we demonstrated a 30.2% reduction in NeuN-positive cells in the IUGR parietal cortex compared to NGs (Figure 5D; *p* = 0.0011). Qualitatively we observed a similar morphology to our recent observations of NeuN-positive cells in the IUGR brain with a smaller size and less defined neuronal labeling pattern (Figure 5B) (Wixey et al., 2019) relative to dense, clear healthy NGs (Figure 5A). Three days of ibuprofen treatment significantly ameliorated the decrease in NeuN-positive cell counts in the parietal cortex of IUGR treated piglets compared to untreated IUGR piglets (Figure 5D) with NeuN-positive counts 23.4% higher in ibuprofen treated IUGR piglets than untreated IUGR piglets (Figure 5D; *p* = 0.0213). There was no significant difference in the number of NeuN-positive neurons between NGs and treated IUGR piglets. Furthermore the NeuN-positive neuronal morphology appeared more full bodied in the ibuprofen treated IUGR piglet brain, resembling the morphology of NeuN-positive neurons in the NG brain (Figure 5C).

### Apoptosis and Cellular Proliferation in the IUGR Piglet Brain Following Ibuprofen Treatment

**FIGURE 6:**
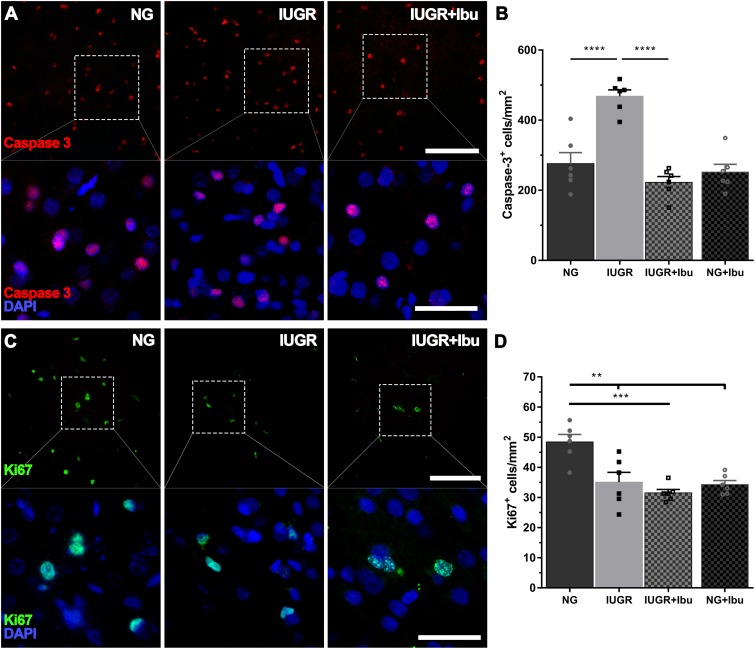
Ibuprofen reduces apoptosis but has does not ameliorate cellular proliferation in IUGR. **(A)** Representative images of apoptotic cells (caspase 3-postive, red) in the parietal cortex of both untreated NG, IUGR, and IUGR+Ibu. **(B)** IUGR displayed higher apoptotic cell counts compared with NG. Ibuprofen significantly reduced the number of Caspase-3 cells in IUGR brains to levels comparable to NG. **(C)** Cellular proliferation was observed using Ki67. **(D)** Ki67 expression was significantly higher in untreated NG compared with all other groups. For **(A,C)** scale bar = 100 μm for low magnification and 50 μm for high magnification. For all data *n* = 6 for each group. Values are presented as mean ± SEM. Two-way ANOVA with Sidak *post hoc* test (^∗∗^*p* < 0.005; ^∗∗∗^*p* < 0.001; ^∗∗∗∗^*p* < 0.0001).

We previously demonstrated an increase in cellular apoptosis and reduction and cellular proliferation (Wixey et al., 2019) at P4 in the IUGR piglet parietal cortex and therefore proceeded to determine whether ibuprofen treatment could ameliorate these changes. In the current study we confirm the increase in caspase-3-positive cell counts in the IUGR piglet parietal cortex compared to NG (*p* < 0.0001; Figure 6A,B). Furthermore, ibuprofen treatment significantly prevented the increase in caspase-3-positive cell counts in the parietal cortex in the IUGR treated animals in comparison to untreated IUGR piglets (*p* < 0.0001; Figure 6A,B). No significant differences in caspase-3-positive cell counts were observed between NG and IUGR treated (*p* = 0.3808) and NG treated (*p* = 0.8811) piglets.

We observed a significant decrease in Ki67-positive cells in the IUGR brains in comparison to NG piglets (*p* = 0.0021; Figure 6C,D), in agreement with our previous findings (Wixey et al., 2019). However, ibuprofen treatment did not ameliorate this reduction in the IUGR piglet. Furthermore, we demonstrate a significant reduction in Ki67-positive cells in both the IUGR ibuprofen treated (*p* = 0.0002) and NG ibuprofen treated (*p* = 0.0012) piglet brains compared to untreated NG piglets (Figure 6C,D).

## Discussion

We present novel evidence that postnatal oral ibuprofen treatment reduces neuroinflammation and neuronal and white matter impairment in the newborn IUGR piglet. A repeated daily dose of ibuprofen for 3 days ameliorated reductions in myelin index, neuronal cell counts, and increases in caspase-3-positive cells at P4. The reductions in numbers of activated microglia, astrogliosis, and expression of a number of proinflammatory cytokines suggest that ibuprofen may reduce neuronal and white matter impairment by hindering the actions of neuroinflammatory mediators during the first few days following birth. These findings reveal a potential new application for ibuprofen to treat brain impairment associated with IUGR in the newborn. As ibuprofen is currently administered to the newborn for other purposes, these findings are encouraging, as there is currently no effective therapeutic intervention available to reduce neurological disorders in the IUGR newborn.

### Brain Impairment in IUGR Newborn Piglets

Clinical MRI studies show reduced gray matter and white volume and structure in human IUGR newborns and in early infancy (Tolsa et al., 2004; Esteban et al., 2010; Padilla et al., 2014), with these disturbances being associated with adverse neurological outcomes in the IUGR infant (Batalle et al., 2012, 2013; Eixarch et al., 2016). At a microscopic level, IUGR animal studies also demonstrate these neuronal and white matter disturbances (Mallard et al., 2000; Guo et al., 2010; Mazur et al., 2010; Alves De Alencar Rocha et al., 2017; Kalanjati et al., 2017; Ruff et al., 2017; Wixey et al., 2019). The current study corroborates these findings demonstrating a reduction in neuronal cell counts and myelination in the newborn IUGR piglet brain. We further demonstrate reduced cellular proliferation and increased cell death in the IUGR piglet brain as previously reported in piglet and rat IUGR animal models (Pham et al., 2015; Wixey et al., 2019) suggesting brains of IUGR newborns are not just in a state of delayed neuronal maturation, but in a state of ongoing cellular injury following birth. Therefore there is a real potential to therapeutically treat, and in doing so, reduce this ongoing injury.

### Dose of Ibuprofen

The dosage and treatment regime used in the current study was adopted from the human clinical situation where ibuprofen is administered to treat patent ductus arteriosus in the preterm neonate (Ohlsson et al., 2015). This dosage is much lower than other neonatal animal studies where high dosages of ibuprofen have been administered daily (50–100 mg/kg/day), that even though are neuroprotective (Carty et al., 2011; Wixey et al., 2012), may be toxic to other organs such as the renal and gastrointestinal tract (De Martino et al., 2017). Ibuprofen is a lipophilic compound that crosses the blood–brain barrier (BBB) (Parepally et al., 2006; Kokki et al., 2007). Oral ibuprofen administration has an excellent absorption rate in the preterm newborn (Barzilay et al., 2012). A pharmacokinetic study of a single dose of oral ibuprofen (10 mg/kg) in 13 preterm infants showed all infants had detectable ibuprofen levels 1 h after administration, with ibuprofen levels peaking around 8 h and remaining plateau until 24 h (Barzilay et al., 2012). Thus, the daily dosage in the current study (20 mg/kg day 1 and 10 mg/kg days 2 and 3) would be sufficient to maintain pharmaceutical levels of ibuprofen throughout the 3 days.

### Oral Ibuprofen Administration Improves IUGR Brain Outcomes

#### Inflammatory Markers

Previous studies have demonstrated ibuprofen’s ability to target neuroinflammation by reducing numbers of activated microglia and levels of proinflammatory cytokines in the P3 HI rat brain (Carty et al., 2011; Wixey et al., 2012). Although these studies used much higher dosages of ibuprofen (50–100/kg/day) for a longer period of time (7 days) than used in the current study, similar responses were observed in the current study with reductions in numbers of activated microglia and proinflammatory cytokines following just 3 days of ibuprofen administration. Ibuprofen treatment has also been shown to have an effect on astrogliosis in an animal model of dementia (Sekiyama et al., 2012). In the current study we demonstrate not only a recovery of the density of astrocytes, but the morphology of the astrocytes in the IUGR treated brain were similar to NGs demonstrating ibuprofen’s effect on the morphology of the astrocytes. Whether this is a direct or indirect effect on these glial cells requires further investigation.

Activated microglia are hallmark features of neuroinflammatory processes that occur after hypoxic events. Increased numbers of activated microglia are associated with brain injury in the HI neonatal rat model (Wixey et al., 2009, 2011b), and increased microglial numbers have been demonstrated in IUGR animal models (Olivier et al., 2007; Black et al., 2015; Pham et al., 2015; Wixey et al., 2019). Selectively blocking microglia (using the tetracycline drug minocycline) decreases not only the inflammatory response but also reduces both neuronal and white matter injury in the neonatal HI rat brain (Carty et al., 2008; Wixey et al., 2011a,b). In the current study we demonstrate a similar response with ibuprofen’s ability to ameliorate the increase in numbers of activated microglia in the IUGR brain. Though activated microglia, broadly speaking, can exist in different forms: M1-like (pro-inflammatory) and M2-like (anti-inflammatory). We can assume the IUGR brain is in a proinflammatory state from the PCR inflammatory panel results and therefore ibuprofen is blocking the proinflammatory microglia rather than the anti-inflammatory microglia, however this remains to be fully elucidated. It is also important to note that complete blockade of microglial activity can exacerbate brain damage as seen in adult and neonatal HI injury models (Lalancette-Hebert et al., 2007; Faustino et al., 2011). Therapeutic interventions specifically blocking M1-like microglia and favoring M2-like microglia could be beneficial to protect the injured brain. Minocycline selectively blocks M1-like microglia and does not affect M2-like microglia expression (Kobayashi et al., 2013). However, minocycline is not the drug of choice to reduce brain injury in the newborn as it can have adverse effects when administered to neonates. Whether ibuprofen has the potential to selectively block M1-like activated microglia requires further investigation.

When microglia become activated they can release large amounts of proinflammatory cytokines which can be toxic to neurons and white matter in the newborn brain. Overexpression of IL-1β is associated with white matter damage in neonatal human brain; with specific co-localization to microglia and astrocytes (Girard et al., 2010). Variability in cytokine response was apparent in the IUGR brain as well as in response to ibuprofen treatment. A greater proportion of proinflammatory cytokines were increased in the IUGR brain with little changes to the anti-inflammatory cytokine expression; demonstrating the IUGR brain may mainly be in a proinflammatory state. This is further evident from our recent study demonstrating expression of IL-1β and TNF-α in neuronal and glial cells in P4 IUGR piglet brains (Wixey et al., 2019). The significant increases we observed in TNF-α, CXCL10, and IL-1β expression in the IUGR brain were alleviated following ibuprofen treatment. TNF-α, CXCL10, and IL-1β are common cytokines involved in the inflammatory response in the neonate. High levels of TNF-α, IL-1β, and CXCL10 are present in amniotic fluid from pregnancies complicated by infection compared to uninfected controls (Scott et al., 2012). An increase in multiple cytokines including TNF-α, IL-1β, CXCL10 are observed in a guinea pig model of IUGR with these increases relating to increased apoptosis and neuronal loss (Guo et al., 2010). A rat neonatal HI study demonstrated similar results to the current study with increases in CXCL10, TNF-α, and IL-1β (Jaworska et al., 2017). The levels of increase were similar with modest increases in TNF-α and IL-1β, and highly significant increases in CXCL10 as we observed. Furthermore, a neuroprotective strategy, sodium butyrate, suppressed the upregulation of CXCL10 at 48 h post-insult. However only at 6 days post-insult did the reduction from treatment occur for IL-1β expression, with no significant effect on TNF-α, although there was a tendency toward a decrease in expression (Jaworska et al., 2017).

Increased levels of TNF-α and IL-1β have been demonstrated in the blood of IUGR infants on day 14 following birth, which are not evident at birth (Mcelrath et al., 2013). IUGR infants tend to have low circulating levels of inflammatory proteins in their blood during the first four postnatal days (Hecht et al., 2011). Furthermore, increases in inflammatory cytokines collected during the first two postnatal weeks are correlated with adverse neurobehavioral outcomes in small for gestational age infants at 2 years (Leviton et al., 2013). Determining whether these increases in inflammatory cytokines are evident in the IUGR piglet blood would be advantageous to determine the potential for a correlation between inflammatory markers in blood and brain. If proven, we could detect and determine the extent of brain injury and thus response to treatment. Furthermore, it is yet to be determined whether the inflammation is originating from the blood or brain. As increases in inflammatory markers are only evident in the IUGR human blood 2 weeks after birth (Mcelrath et al., 2013) it is plausible inflammation is originating from the brain and these inflammatory markers may be released into the blood due to BBB breakdown; as BBB disruption occurs in the IUGR brain (Castillo-Melendez et al., 2015). Future studies should include a focus on protein levels of cytokines in both the blood and cerebral spinal fluid to assess both the brain environment and other factors that may be contributing to this inflammatory state in the IUGR newborn.

#### Neuronal and White Matter Impairment

We have previously shown sustained neuronal and white matter impairment in the IUGR brain up to postnatal day 7 (Kalanjati et al., 2017; Wixey et al., 2019). In the current study we found that 3 days of ibuprofen treatment from day of birth was sufficient to alleviate the decrease in neuronal cell counts and myelination disruption in the IUGR piglet brain, therefore ceasing this injurious pattern. Whether 3 days of treatment is enough to evoke long lasting neuroprotection remains to be determined. Yet the rapid neuronal and white matter recovery observed in the current study suggests administering ibuprofen on day of birth may afford not only neuroprotection, but other mechanisms are at play. A body of evidence states alterations in myelination in the IUGR brain arise due to stalling of oligodendroglial cell maturation (Tolcos et al., 2011). This block in maturation occurs at a premyelinating stage of oligodendrocyte development, resulting in reduced density of mature myelinating oligodendrocytes. Whether ibuprofen treatment can ‘unblock’ the oligodendroglial cell maturation arrest in the IUGR brain is unknown. However a study in the neonatal HI rat model shows 1 week of ibuprofen treatment alleviated the loss of O4-positive premyelinating oligodendrocytes and O1-positive immature oligodendrocytes progenitor cells (Carty et al., 2011). Therefore the possibility of a similar response in the IUGR brain may be likely. Furthermore, whether a similar phenomenon is apparent for the neuronal population, i.e., a developmental stall in the immature neurons may occur in the IUGR brain and ibuprofen releases this effect, may also be plausible. Further immunohistochemistry studies on oligodendrocyte lineage markers, immature neuronal markers and mechanistic studies would be useful to decipher the role of ibuprofen in neuronal and white matter recovery.

Exploring ibuprofen’s effects on Ki67-cellular proliferation would also be useful to determine whether this significant reduction following treatment in both the IUGR and NG piglets may have a detrimental impact in the long term. No other adverse outcomes following treatment were observed in these piglets therefore this decrease may not exert a physiological impact. It would be beneficial to determine the cell type of these proliferative cells as we have previously shown very rare colocalization of Ki67-positive cells with NeuN-positive cells (Wixey et al., 2019). We have unpublished evidence that Ki67 co-localizes with Iba-1-positive microglial cells in the parietal cortex. The decrease in Ki67-positive cells we observed in the current study may be due to a decrease in the activated microglia in these treated animals. However, whether this cell type is the population decreasing in the current study requires in depth exploration with double labeling of multiple cellular phenotypes. This will determine whether this decrease in Ki67-cellular proliferation is or is not a concerning aspect to treatment. Examining other regions of the brain rich in proliferative cells (such as the subventricular zone) and later time points may explain our current findings. Furthermore, follow up studies examining another common marker for proliferating cells (BrdU – which detects cells during DNA replication) may answer these questions. Yet, just 3 days of ibuprofen treatment was enough to cease the cycle of cellular injury. The highly significant increases in caspase-3-positive cell counts were completely ameliorated in the IUGR brain following ibuprofen treatment. Interestingly, there is emerging evidence that ibuprofen directly (independent of COX actions) inhibits caspases and cell death (Aranda et al., 2017; Smith et al., 2017). Determining which caspase-3-positive cell types were affected in the IUGR brain following treatment would be advantageous to determine in more detail, ibuprofen’s response in the brain. These cells may be a combination of microglia, astrocytes and neurons, however further detailed investigations are warranted.

### Putative Mechanisms of Ibuprofen Protection of IUGR Brain Impairment

While the role of ibuprofen as a COX inhibitor is well-established, other targets may contribute to its neuroprotective effects. Recent reports have demonstrated ibuprofen’s additional targets such as caspases, Rho activity, and peroxisome proliferator-activated receptor-γ (PPAR-γ) (Heneka et al., 1999; Landreth and Heneka, 2001; Asanuma and Miyazaki, 2006; Rocha et al., 2015; Smith et al., 2017).

Ibuprofen is a potent agonist of the PPAR-γ. PPAR-γ plays a role in the modulation of immune responses through suppression of proinflammatory gene expression (Villapol, 2018). Ibuprofen has been shown to exert neuroprotective effects by inhibiting gene expression of inflammatory mediators (microglia, TNF-α, IL-6, and iNOS) through the activation of PPAR-γ (Heneka et al., 1999; Landreth and Heneka, 2001; Asanuma and Miyazaki, 2006). Ibuprofen has also been found to be an astroglial inhibitor, whereby decreasing GFAP and iNOS expression (Lim et al., 2000; Heneka et al., 2005). Ibuprofen administration diminishes reactive astrogliosis in dementia animal models (Lim et al., 2000; Heneka et al., 2005; Sekiyama et al., 2012). Its action on astrocytes is seemingly dependent on RhoA activity (Rocha et al., 2015). RhoA is known to play a role in astrocyte reactivity, and *in vitro* inhibition of RhoA with ibuprofen treatment diminishes astrogliosis (Rocha et al., 2015). Rho activation also plays a critical role in limiting axonal regeneration following CNS injuries and therapeutically inhibiting Rho via ibuprofen treatment is an important target for axonal repair in injured CNS neurons (Madura et al., 2011). A recent finding demonstrates physiologically relevant concentrations of ibuprofen impedes caspase catalysis, reducing inflammation and cell death both *in vitro* and *in vivo* (Smith et al., 2017). The study showed that caspase inhibition is COX independent and represents a new anti-inflammatory target as caspases play a pivotal role in inflammation and cell death (Smith et al., 2017). It is likely that both COX and caspase pathways are simultaneously modulated following ibuprofen treatment, each contributing to the anti-inflammatory mechanism. As we demonstrated a significant decrease in the number of casapase-3 cells in the IUGR brain, this could be due to a direct effect of ibuprofen on apoptosis and inflammation.

### Limitations

Although the results from the current study are promising, there are a few limitations to this study. The piglet brains were examined immediately after treatment ceased and therefore we were unable to adequately assess potential long-term adverse effects or long-term benefits of the 3 days of ibuprofen treatment. Surviving the piglets for a longer time period after treatment has ceased would be beneficial to explore this avenue of research. Determining the type of proliferating and apoptotic cells would also prove beneficial, especially in regard to the treatment response of the proliferating cells. There are multiple cellular phenotypes in the brain, however further examination of oligodendrocyte lineage markers and immature neuronal markers would assist with understanding the major neurodevelopmental networks of the IUGR newborn brain and their response to treatment.

## Conclusion

Currently, there are limited treatments to prevent neurological impairment in the IUGR infant. Understanding the impact of inflammation in the IUGR brain is critical for the development of therapies to improving neurodevelopmental outcomes in IUGR newborns. As evidenced in this study, ibuprofen may be effective at protecting the IUGR newborn brain and be a promising therapeutic option. Long-term studies testing the efficacy of early ibuprofen treatment would assist in determining the potential clinical application of ibuprofen as an intervention for protecting the newborn brain. It is notable that in an adult rodent model, 6 days of ibuprofen treatment protects neurons from ischemic-induced injury for up to 4 weeks post-insult (Park et al., 2005). Exploring interventions that specifically target inflammatory processes could not only reduce white matter and neuronal injury in the brain but may provide neuroprotection through effects on central and systemic inflammation.

## Ethics Statement

This study was carried out in accordance with the recommendations of the ARRIVE guidelines. The protocol was approved by The University of Queensland Animal Ethics Committee (MED/UQCCR/132/16/RBWH).

## Author Contributions

JW was involved in attaining funding, experimental designs, conducting animal experiments, critical revision, and drafting of the manuscript. KS and RP conducted the animal experiments, laboratory aspects, and collated and analyzed the data. KL undertook Luxol Fast Blue staining, analysis, and interpretation. PC was involved in attaining funding, critical revision, and editing the manuscript. SB was involved in attaining funding, critical revision, and editing the manuscript. KC was involved in attaining funding and responsible for all laboratory aspects of the project, data analysis, interpretation, and editing the manuscript. All authors read and approved the final manuscript.

## Conflict of Interest Statement

The authors declare that the research was conducted in the absence of any commercial or financial relationships that could be construed as a potential conflict of interest.
